# Association between Promoter Hypomethylation and Overexpression of Autotaxin with Outcome Parameters in Biliary Atresia

**DOI:** 10.1371/journal.pone.0169306

**Published:** 2017-01-04

**Authors:** Wanvisa Udomsinprasert, Nakarin Kitkumthorn, Apiwat Mutirangura, Voranush Chongsrisawat, Yong Poovorawan, Sittisak Honsawek

**Affiliations:** 1 Department of Biochemistry, Faculty of Medicine, Chulalongkorn University, King Chulalongkorn Memorial Hospital, Thai Red Cross Society, Bangkok, Thailand; 2 Department of Oral and Biology, Faculty of Dentistry, Mahidol University, Bangkok, Thailand; 3 Center of Excellence in Molecular Genetics of Cancer and Human Diseases, Department of Anatomy, Faculty of Medicine, Chulalongkorn University, Bangkok, Thailand; 4 Center of Excellence in Clinical Virology, Department of Pediatrics, Faculty of Medicine, Chulalongkorn University, Bangkok, Thailand; Texas A&M University, UNITED STATES

## Abstract

**Objective:**

Biliary atresia (BA) is a progressive fibroinflammatory liver disease. Autotaxin (ATX) has a profibrotic effect resulting from lysophosphatidic acid activity. The purpose of this study was to examine *ATX* expression and *ATX* promoter methylation in peripheral blood leukocytes and liver tissues from BA patients and controls and investigate their associations with outcome parameters in BA patients.

**Methods:**

A total of 130 subjects (65 BA patients and 65 age-matched controls) were enrolled. DNA was extracted from circulating leukocytes and liver tissues of BA patients and from and age-matched controls. *ATX* promoter methylation status was determined by bisulfite pyrosequencing. *ATX* expression was analyzed using quantitative real-time polymerase chain reaction and enzyme-linked immunosorbent assay.

**Results:**

Decreased methylation of specific CpGs were observed at the *ATX* promoter in BA patients. Subsequent analysis revealed that BA patients with advanced stage had lower methylation levels of *ATX* promoter than those with early stage. *ATX* promoter methylation levels were found to be associated with hepatic dysfunction in BA. In addition, *ATX* expression was significantly elevated and correlated with a decrease in *ATX* promoter methylation in BA patients compared to the controls. Furthermore, promoter hypomethylation and overexpression of *ATX* were inversely associated with jaundice status, hepatic dysfunction, and liver stiffness in BA patients.

**Conclusion:**

Accordingly, it has been hypothesized that *ATX* promoter methylation and *ATX* expression in peripheral blood may serve as possible biomarkers reflecting the progression of liver fibrosis in postoperative BA. These findings suggest that the promoter hypomethylation and overexpression of *ATX* might play a contributory role in the pathogenesis of liver fibrosis in BA.

## Introduction

Biliary atresia (BA) is a devastating cholestatic liver disorder of uncertain etiology in neonates and manifests as impaired liver function and fibroinflammatory obliterative cholangiopathy of both the intrahepatic and extrahepatic bile ducts [1]. BA patients initially develop neonatal jaundice due to hepatic cholestasis and progress to liver fibrosis, which leads to cirrhosis [2]. Since no medical therapies exist, sequential treatment by surgical hepatoportoenterostomy or Kasai procedure and liver transplantation is the only option for therapy in most affected children due to complications of cirrhosis [3]. Although the precise pathogenesis of BA has yet to be determined, multiple theories exist regarding the etiology of BA, including viral infection, inflammation, bile duct proliferation, and fibrogenesis [4].

Autotaxin (ATX), a secreted glycoprotein, belongs to the ectonucleotide pyrophosphatase/phosphodiesterase (ENPP) enzyme family. ATX exhibits a unique lysophospholipase D (LPD) activity, converting lysophosphatidylcholine (LPC) to lysophosphatidic acid (LPA) [5]. LPA acts via activation of at least six different G-protein-coupled receptors to influence a number of biological processes [6]. Both ATX and LPA are considered to be involved in the development of liver fibrosis and elevated serum ATX was associated with liver fibrosis in cirrhotic patients and hepatocellular carcinoma (HCC) patients [7, 8]. LPA can induce hepatic stellate cell (HSC) proliferation, stimulate their contraction, and inhibit their apoptosis [9]. Liver fibrosis is the excessive accumulation of extracellular matrix (ECM) proteins that occurs in most types of chronic liver diseases. HSCs were recognized as the main matrix-producing cells in the liver. In continuously injured livers, HSCs are activated and transdifferentiate into myofibroblasts, resulting in the production of abundant extracellular matrices and profibrogenic cytokines including ATX-mediated LPA. Serum ATX has been proposed as a marker for liver fibrosis [10]. Recent studies have also suggested a connection between liver fibrosis and circulating LPA, and serum ATX was elevated in chronic hepatitis C patients [11, 12]. Importantly, the ATX-LPA axis has been shown to be upregulated in human HCC, suggesting that ATX possibly plays an important role in inflammation-related liver fibrogenesis [13, 14]. Given that elevated ATX levels contribute to the pathogenesis of liver fibrosis in BA, we hypothesized that the hypomethylation of the *ATX* promoter region could upregulate *ATX* expression in BA patients.

It has been demonstrated that there is a possible link between epigenetic regulation and the etiologic mechanism of intrahepatic bile duct defects in BA [15]. Methylation of cytosine–guanine dinucleotide (CpG) residues catalysed by DNA methyltransferases (DNMTs) within the promoter and enhancer regions leads to suppression of gene expression and DNA methylation alterations can be elicited by viruses and genetic defects [16–18]. DNA hypomethylation may result in the development of several autoimmune disorders, such as systemic lupus erythematosus and rheumatoid arthritis [19, 20]. Recent evidence indicates that alterations to epigenetic DNA methylation patterns contribute to the pathogenesis of BA. The hypermethylation of promoter regulatory elements contributes to the lower CD11a expression in T lymphocytes of BA patients [21]. However, the hypomethylation of the interferon gamma (IFN-γ) gene promoter may be responsible for increased IFN-γ expression in BA infants [22]. These findings suggest the significance of aberrant DNA methylation in the development of BA.

Until now, there have been no reports regarding the role of *ATX* promoter methylation and expression in BA patients. We recently reported that elevated serum ATX was significantly associated with hepatic dysfunction and severity in BA patients [23]. The purpose of this study was to investigate promoter methylation status and expression of *ATX* in peripheral blood leukocytes and liver tissues from BA patients compared with controls. We also explored whether *ATX* promoter methylation and expression were associated with the clinical parameters of BA patients.

## Materials and Methods

### Study population

The study protocol was approved by the Institutional Review Board of the Faculty of Medicine, Chulalongkorn University (IRB number 279/57). This study was conducted in accordance with the ethical standards outlined in the 1975 Declaration of Helsinki. All participants, parents, or legal guardians were fully informed regarding the study protocol and procedures prior to participating in the study. Written informed consent was obtained from all patients and from parents or legal guardians of patients younger than 18 years of age.

This cross-sectional study evaluated 65 BA patients, 65 age-matched unaffected volunteers. BA patients were diagnosed by intra-operational cholangiography and were surgically treated with Kasai portoenterostomy at King Chulalongkorn Memorial Hospital, Bangkok, Thailand. Patients who had undergone liver transplantation were excluded. Unaffected volunteers who attended the Well Baby Clinic at King Chulalongkorn Hospital for vaccination who had normal physical findings and no underlying diseases were included. We stratified BA patients according to liver stiffness values into two groups: non-fibrotic BA (<9.7 kPa, n = 22) and fibrotic BA (>9.7 kPa, n = 43). We also classified BA patients according to serum total bilirubin (TB) into either the non-jaundice group (TB<2 mg/dl, n = 41) or the persistent jaundice group (TB≥2 mg/dl, n = 24). Based on the severity of hepatic injury (aspartate aminotransferase, AST value), BA patients were divided into either the early-stage group (AST<100 IU/l, n = 36) or late-stage group (AST≥100 IU/l, n = 29).

### Liver tissue samples

Liver tissue samples of 15 BA patients (8 females and 7 males; age range 1–4 months; mean 66 days) who underwent Kasai operations and 5 non-BA patients who underwent liver biopsies with no signs of fibrosis were obtained at the Department of Surgery, King Chulalongkorn Memorial Hospital, between 2001 and 2006. The non-BA patients who had no clinical jaundice, served as controls. All non-BA patients underwent exploratory laparotomy as the therapeutic treatment for their diseases. Liver biopsies in this group of patients were an additional procedure and were required for medical reasons. All samples were obtained with the families’ consent.

### Assessment of clinical outcome

Venous blood samples were drawn from each subject in ethylenediaminetetraacetic acid and clot blood tubes for routine laboratory tests, including TB, AST, alanine aminotransferase (ALT), alkaline phosphatase (ALP), and albumin. All of the immediately aforementioned tests were performed on a Roche Hitachi 912 Chemistry Analyzer (Roche Diagnostics, Basel, Switzerland). Assessment of liver stiffness by transient elastography was performed using a Fibroscan (EchoSens, Paris, France). Briefly, measurements were performed by placing the Fibroscan transducer probe on the intercostal space at the area of the right lobe of the liver. Measurements were then performed until 10 validated results were obtained with a success rate of at least 80%. The median value from 10 validated scores represented the elastic modulus measurement of the liver, which was expressed in kilopascals (kPa).

### Bisulfite pyrosequencing of *ATX* promoter

Genomic DNA was isolated from peripheral blood leukocytes and liver tissue samples using DNA isolation kits (GE Healthcare, Buckinghamshire, UK and Vivantis, Selangor Darul Ehsan, Malaysia, respectively). Our assay was designed to examine methylation levels at four CpG sites within the *ATX* promoter. Quantitative DNA methylation analysis of each CpG was measured on bisulfite-treated DNA using highly quantitative analysis based on polymerase chain reaction (PCR) pyrosequencing. Briefly, extracted DNA (50 ng; concentration: 2.5 ng/μl) was bisulfite converted using an EZ DNA Methylation-Gold^™^ Kit (Zymo Research Corporation, Orange, CA, USA). Each 30 μl PCR reaction contained 10X PCR buffer, 200 mM dNTPs, 0.2 mM primers, 0.5 U HotStar Taq DNA polymerase (Qiagen, Inc., San Diego, CA, USA), and 5 ng of bisulfite-treated DNA. The polymerase was activated by incubation at 95°C for 15 min, followed by 40 cycles of 95°C for 1 min, 56°C for 1 min, and 72°C for 1 min. The reaction was then allowed to develop for 7 min at 72°C. The primers used to measure *ATX* promoter methylation were as follows: forward primer 5’-TAGGTATTGTAGGGGGTGGGAA-3’; reverse primer biotinylated-5’-ACCTTTAACAAAACACACACATAACC-3’; and, sequencing primer 5’-GGGTGGGAATGTGGA-3’. PCR products (20 μl) were purified and analyzed in the PyroMark MA System (Pyrosequencing, Inc., Westborough, MA, USA) and methylation data from the amplified regions were analyzed by Pyro Q-CpG software 1.0.6. The degree of methylation was expressed for each CpG site as a percent of methylated cytosine.

### Quantitative real-time polymerase chain reaction (QPCR)

To evaluate mRNA expression of *ATX* in BA patients and controls using real-time PCR, total RNA from peripheral blood leukocytes and liver tissue samples was extracted by Trizol (Invitrogen, Carlsbad, CA, USA), with cDNA reverse transcribed by a Roche Transcriptor cDNA synthesis kit (Roche, Branchburg, NJ, USA). Real-time PCR was performed using QPCR Green Mastermix HRox (biotechrabbit GmbH, Hennigsdorf, Germany) on a StepOnePlus Real Time PCR system (Applied Biosystems, Inc., Foster City, CA, USA). The primers used for *ATX*, *DNA methyltransferase 1 (DNMT1)*, and glyceraldehyde 3-phosphate dehydrogenase (GAPDH) amplification were as follows: *ATX* forward primer 5’-CGTGGCTGGGAGTGTACTAA-3’; *ATX* reverse primer 5’AGAGTGTGTGCCACAAGACC-3’, as previously described [8]; *DNMT1* forward primer 5’- CAGGCCCAATGAGACTGACA-3’, and *DNMT1* reverse primer 5’- GTGGGTGTTCTCAGGCCTGTAG-3’; *GAPDH* forward primer 5’-GTGAAGGTCGGAGTCAACGG-3’, and *GAPDH* reverse primer 5’-TCAATGAAGGGGTCATTGATGG-3’. The real-time PCR conditions were performed as follows: (initial step) 95°C for 10 min, followed by 40 cycles of 95°C for 15 sec, and then 60°C for 1 min. The relative mRNA expression of *ATX* and *DNMT1* was normalized to GAPDH as an internal control and was determined using the 2^-ΔΔCt^ method.

### Enzyme-linked immunosorbent assay (ELISA)

Total protein from liver tissue was extracted by a RIPA assay (Cell Signaling Technology, Inc., Danvers, MA, USA), which contained a protease inhibitor (Merck Millipore, Darmstadt, Germany). Serum and protein lysate ATX concentrations were determined using a commercially available sandwich enzyme-linked immunosorbent assay development kit (R&D Systems, Inc., Minneapolis, MN, USA).

### Statistical analysis

All statistical analyses were performed using SPSS version 22.0 (SPSS, Inc., Chicago, IL, USA). The statistical significance between demographic data of patients and controls was compared by unpaired Student’s *t*-test. Given that *ATX* promoter methylation data were not normally distributed, nonparametric tests were applied as follows: Mann-Whitney *U* test for comparing DNA methylation levels between groups and Kruskal-Wallis H test for continuous variables. The association of methylation changes as well as *ATX* expression and clinical parameters were detected using multivariate linear regression analysis. Correlations between DNA methylation, mRNA expression, protein concentration, and clinical parameters were evaluated using Spearman’s correlation coefficient (*r*). Receiver operating characteristic (ROC) curve and the area under the ROC curve (AUC) were calculated to assess the feasibility of using *ATX* methylation status as a possible parameter in discriminating BA patients from controls. Data were shown as a mean±standard error of the mean. *P*-values less than 0.05 were considered statistically significant for all analyses.

## Results

### Characteristics of study participants

The baseline clinical characteristics of study participants are summarized in Table 1. A total of 130 subjects (65 BA patients and 65 age-matched healthy controls) were recruited in the current study. There were no significant differences in age or gender between BA patients and healthy controls. As expected, clinical parameters, including liver stiffness values, AST, and ALT were significantly higher in BA patients than those in controls (*P*<0.0001).

**Table 1:** Clinical characteristics of study participants.

Clinical characteristics	BA patients (n = 65)	Healthy controls (n = 65)	*P*-value
**Age (years)**	8.55±0.52	8.27±0.69	0.92
**Gender (female:male)**	35:30	32:33	0.87
**Liver stiffness (kPa)**	25.30±2.71	4.01±0.19	<0.0001*
**TB (mg/dl)**	2.10±0.41	-	NA
**AST (IU/l)**	105.07±8.94	26.66±0.82	<0.0001*
**ALT (IU/l)**	93.97±9.19	9.24±0.65	<0.0001*
**ALP (IU/l)**	368.17±27.61	-	NA
**Albumin (mg/dl)**	4.24±0.10	-	NA

Data are presented as mean ± standard error of the mean, unless otherwise specified

*Differences in descriptive data are considered significant at *P*-value less than 0.05 (two-tailed)

Abbreviations: BA = biliary atresia; TB = total bilirubin; AST = aspartate aminotransferase; ALT = alanine aminotransferase; ALP = Alkaline phosphatase; NA = not available

### *ATX* promoter hypomethylation in peripheral blood leukocytes

**Fig 1:**
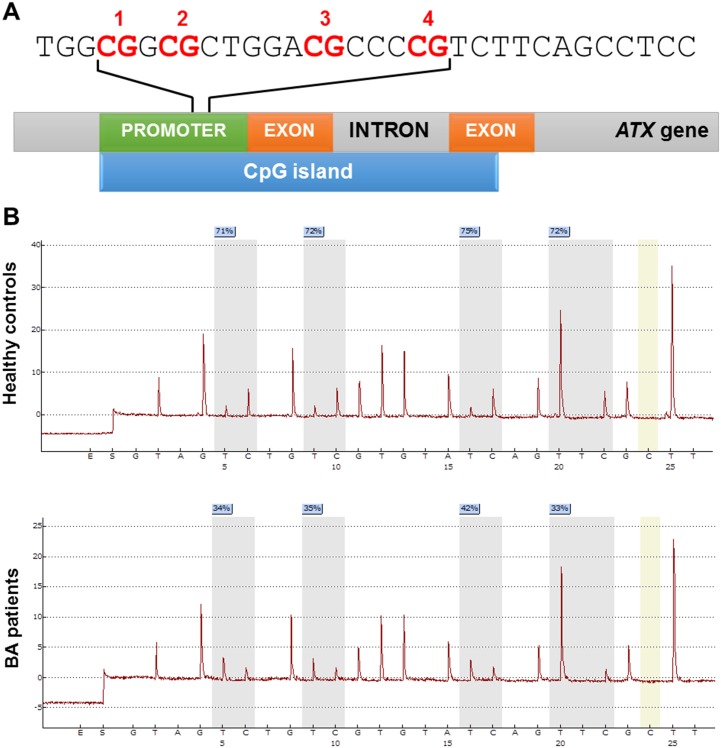
DNA methylation at four CpG islands within *ATX* gene promoter. (A) Schematic diagram representing CpG islands at the *ATX* promoter showing the four CpG sites. (B) Pyrosequencing results in healthy controls and BA patients.

**Fig 2:**
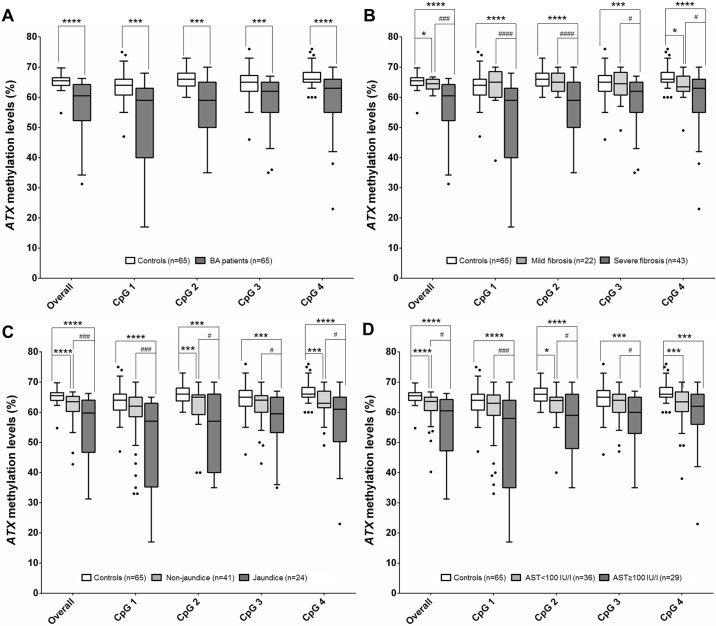
Box-plot illustrating methylation levels of the *ATX* promoter in peripheral blood leukocytes of subjects among different groups. (A) Methylation levels of the *ATX* promoter at four CpG sites in BA patients and healthy controls. (B) Methylation levels of *ATX* promoter at four CpG sites in BA subgroups, including mild fibrosis (F0-F2) and severe fibrosis (F3-F4). (C) Methylation levels of the *ATX* promoter at four CpG sites in patients with and without jaundice. (D) Methylation levels of *ATX* promoter at four CpG sites in BA patients according to severity of hepatic damage (AST values). *P<0.05, **P<0.01, ***P<0.001, ****P<0.0001 vs control group and ^#^*P*<0.05, ^##^*P*<0.01, ^###^*P*<0.001, ^####^*P*<0.0001 for comparisons between BA subgroups.

To explore promoter methylation of *ATX* in BA, we first verified promoter methylation in peripheral blood leukocytes in BA patients and unaffected volunteers. Fig 1 illustrates a schematic representation of four CpG sites within the *ATX* gene promoter region. Overall, methylation levels at the *ATX* promoter were significantly lower in the BA group than healthy controls (*P*<0.0001). Similarly, BA patients demonstrated significantly reduced methylation levels when compared to healthy controls across four CpG sites, as follows: CpG 1: *P* = 0.0029, CpG 2: *P* = 0.0005, CpG 3: *P* = 0.0057, and CpG 4: *P*<0.0001 (Fig 2A).

In analyses stratified by disease severity, we classified BA patients according to fibrosis, jaundice status, and hepatic dysfunction marker (AST value) (Table 2). Overall, decreased methylation levels of the *ATX* promoter were detected in advanced BA patients with fibrosis, persistent jaundice, and late stage hepatic dysfunction as compared with those in early stage (*P* = 0.0003, *P* = 0.0077, and *P* = 0.023, respectively) (Fig 2B–2D).

**Table 2:** Characteristics of biliary atresia patients among different groups according to fibrosis stage, jaundice status, and AST value.

	BA patients (n = 65)								
	Mild fibrosis	Severe fibrosis	*P*-value	Non-jaundice	Jaundice	*P*-value	Low AST	High AST	*P*-value
	(F0-F2, n = 22)	(F3-F4, n = 43)		(TB<2 mg/dl, n = 41)	(TB≥2 mg/dl, n = 24)		(<100 IU/l, n = 36)	(≥100 IU/l, n = 29)	
**Age (years)**	8.30±0.81	8.63±0.65	0.77	8.19±0.60	9.25±4.30	0.34	8.35±0.73	8.72±0.71	0.72
**Gender (female:male)**	14:8	21:22	0.11	23:18	12:12	0.79	20:16	15:14	0.81
**Liver stiffness (kPa)**	7.03±0.33	35.18±3.28	<0.0001*	14.70±1.50	51.89±4.54	<0.0001*	14.12±2.16	40.64±4.19	<0.0001*
**TB (mg/dl)**	0.40±0.04	2.92±0.56	<0.0001*	0.62±0.070	5.47±0.95	<0.0001*	0.64±0.11	3.89±0.77	<0.0001*
**AST (IU/l)**	43.05±5.95	137.40±9.99	<0.0001*	79.09±9.70	170.05±8.93	<0.0001*	51.12±4.01	174.79±8.39	<0.0001*
**ALT (IU/l)**	44.40±7.24	119.31±11.25	<0.0001*	86.35±12.43	115.05±8.38	0.060	53.68±6.04	145.50±13.78	<0.0001*
**ALP (IU/l)**	194.35±25.88	460.49±31.10	<0.0001*	306.83±32.04	520.00±35.48	<0.0001*	262.85±27.21	503.32±38.76	<0.0001*
**Albumin (g/dl)**	4.41±0.27	4.16±0.09	0.27	4.40±0.14	3.91±0.09	0.018*	4.48±0.16	3.98±0.10	0.011*

Data are presented as mean ± standard error of the mean, unless otherwise specified

*Differences in descriptive data are considered significant at *P*-value less than 0.05 (two-tailed)

Abbreviations: BA = biliary atresia; TB = total bilirubin; AST = aspartate aminotransferase; ALT = alanine aminotransferase; ALP = Alkaline phosphatase; NA = not available

Additionally, we observed a negative correlation between *ATX* promoter methylation levels and liver stiffness, TB, AST, and ALP (*r* = -0.43, *P* = 0.001; *r* = -0.31, *P* = 0.015; *r* = -0.41, *P* = 0.001; and, *r* = -0.35, *P* = 0.006, respectively). There was a positive association between *ATX* promoter methylation and serum albumin (*r* = 0.37, *P* = 0.009) (Table 3). For each of the four CpG sites, methylation of the *ATX* promoter across the three CpG sites (CpG 1, CpG 2, and CpG 3) was inversely correlated with liver stiffness, TB, AST, ALT, and ALP. *ATX* promoter methylation levels were found to be positively associated with serum albumin. No relationship between *ATX* methylation at the CpG 4 residue and clinical outcome was observed in BA patients (Table 3).

**Table 3:** Correlations between methylation levels of CpG islands at the *ATX* promoter and clinical parameters in BA patients.

Clinical characteristics	Spearman's rho correlation	CpG islands within the *ATX* promoter				
		Overall	CpG 1	CpG 2	CpG 3	CpG 4
**Age (years)**	Coefficient (*r*)	0.08	0.11	0.13	-0.08	-0.02
	*P-*value	0.52	0.38	0.30	0.52	0.85
**Liver stiffness (kPa)**	Coefficient (*r*)	-0.43	-0.50	-0.40	-0.35	-0.24
	*P-*value	0.001*	<0.0001*	0.001*	0.005*	0.060
**TB (mg/dl)**	Coefficient (*r*)	-0.31	-0.36	-0.30	-0.26	-0.18
	*P-*value	0.015*	0.004*	0.020*	0.042*	0.16
**AST (IU/l)**	Coefficient (*r*)	-0.41	-0.48	-0.40	-0.35	-0.25
	*P-*value	0.001*	<0.0001*	0.002*	0.006*	0.056
**ALT (IU/l)**	Coefficient (*r*)	-0.14	-0.22	-0.14	-0.14	0.02
	*P-*value	0.29	0.085	0.30	0.30	0.88
**ALP (IU/l)**	Coefficient (*r*)	-0.35	-0.41	-0.33	-0.26	-0.24
	*P-*value	0.006*	0.001*	0.011*	0.049*	0.066
**Albumin (mg/dl)**	Coefficient (*r*)	0.37	0.38	0.30	0.29	0.38
	*P-*value	0.009*	0.007*	0.038*	0.040*	0.007*

*Correlation is considered statistically significant at *P*-value less than 0.05 (two-tailed)

Abbreviations: BA = biliary atresia; TB = total bilirubin; AST = aspartate aminotransferase; ALT = alanine aminotransferase; ALP = Alkaline phosphatase

### Overexpression of *ATX* mRNA in peripheral blood leukocytes

**Fig 3:**
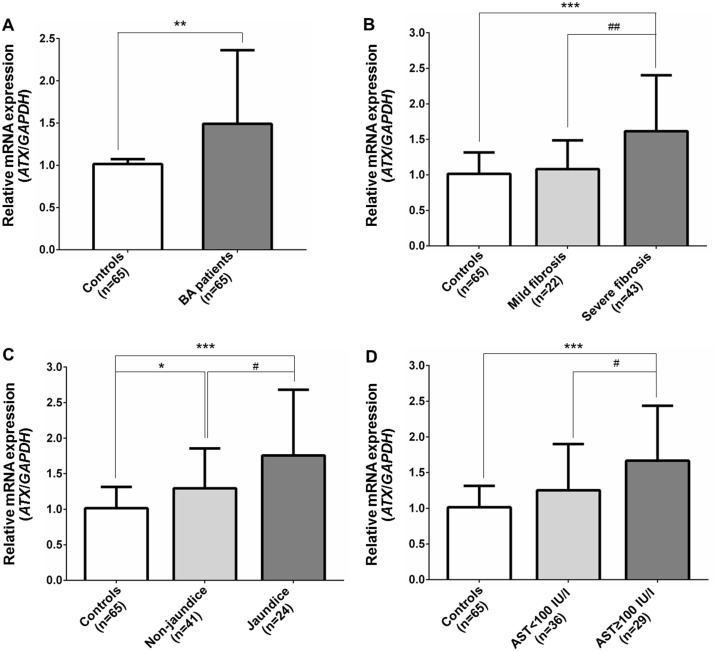
*ATX* mRNA expression in peripheral blood leukocytes of subjects among different groups. (A) Relative *ATX* mRNA expression in BA patients and healthy controls. (B) Relative *ATX* mRNA expression in BA patients with mild and severe fibrosis. (C) Relative *ATX* mRNA expression in BA patients with and without jaundice. (D) Relative *ATX* mRNA expression in early and late stage of hepatic dysfunction in BA patients. Expression was normalized using *GAPDH* as an internal control. Data are expressed as mean and standard deviation. *P<0.05, **P<0.01, ***P<0.001 vs control group and ^#^*P*<0.05, ^##^*P*<0.01 for comparisons between BA subgroups.

*ATX* mRNA expression in peripheral blood leukocytes was significantly elevated in BA patients, as compared with healthy controls (*P* = 0.0096) (Fig 3A). When disease severity was measured, advanced BA patients (severe fibrosis, jaundice, and a high AST value) had significantly higher relative *ATX* mRNA expression than early stage BA patients with mild fibrosis, non-jaundice, and a low AST value (*P* = 0.0056, *P* = 0.015, and *P* = 0.022, respectively) and also healthy controls (*P* = 0.0003, *P* = 0.0003, and *P* = 0.0001, respectively) (Fig 3B–3D).

Subsequent analysis demonstrated positive relationships between the level of *ATX* mRNA expression and clinical parameters including liver stiffness (*r* = 0.43, *P* = 0.001), TB (*r* = 0.49, *P*<0.0001), AST (*r* = 0.36, *P* = 0.005), ALT (*r* = 0.35, *P* = 0.006), and ALP (*r* = 0.47, *P*<0.0001) of BA patients. However, there was no significant correlation between relative *ATX* expression and albumin. We used linear regression to adjust these associations for confounding factors and revealed that upregulation of *ATX* expression was found to be associated with the severity of liver stiffness (β coefficient: 0.012, 95% CI: 0.004 to 0.021, *P* = 0.006), AST values (β coefficient: 0.04, 95% CI: 0.002 to 0.007, *P* = 0.002), and ALP values (β coefficient: 0.001, 95% CI: 0.000 to 0.002, *P* = 0.006) (Table 4).

**Table 4:** Spearman's correlation and multivariate linear regression analysis of *ATX* relative expression estimates.

Variables	Relative mRNA expression (*ATX*/*GAPDH*)			
	Spearman's rho correlation		Linear regression^a^	
	Coefficient (*r*)	*P*-value	β coefficient (95% CI)	*P*-value
**Age (years)**	0.07	0.61	0.013 (-0.032 to 0.058)	0.57
**Liver stiffness (kPa)**	0.43	0.001*	0.012 (0.004 to 0.021)	0.006*
**TB (mg/dl)**	0.49	<0.0001*	0.15 (-0.025 to 0.094)	0.25
**AST (IU/l)**	0.36	0.005*	0.04 (0.002 to 0.007)	0.002*
**ALT (IU/l)**	0.35	0.006*	0.02 (-0.001 to 0.005)	0.13
**ALP (IU/l)**	0.47	<0.0001*	0.001 (0.000 to 0.002)	0.006*
**Albumin (g/dl)**	-0.19	0.20	-0.033 (-0.33 to 0.26)	0.83
***ATX* methylation levels (%)**				
** Overall**	-0.47	<0.0001*	-0.053 (-0.072 to -0.035)	<0.0001*
** CpG 1**	-0.48	<0.0001*	-0.032 (-0.044 to -0.020)	<0.0001*
** CpG 2**	-0.52	<0.0001*	-0.050 (-0.067 to -0.033)	<0.0001*
** CpG 3**	-0.32	0.011*	-0.049 (-0.069 to -0.028)	<0.0001*
** CpG 4**	-0.27	0.030*	-0.043 (-0.066 to -0.019)	0.001*

*Correlation is considered statistically significant at *P*-value less than 0.05 (two-tailed).

^a^The coefficient is adjusted for age and gender.

Abbreviations: TB = total bilirubin; AST = aspartate aminotransferase; ALT = alanine aminotransferase; ALP = Alkaline phosphatase

### Elevated serum ATX levels

The mean ATX concentration in BA patients was significantly higher than that in healthy controls (*P* = 0.012), consistent with evidence from our recent study that serum ATX concentrations were elevated in BA patients [23]. Furthermore, a positive association between relative mRNA expression and serum ATX was observed in BA individuals (*r* = 0.44, *P*<0.0001).

### Correlation between *ATX* methylation, its expression, and protein levels

We investigated associations between changes in DNA methylation, mRNA expression, and circulating protein levels of ATX in BA. The relative *ATX* mRNA expression and serum ATX were both inversely correlated with overall *ATX* methylation levels (*r* = -0.47, *P*<0.0001 and *r* = -0.55, *P*<0.0001, respectively). Using linear regression model, we observed negative associations between *ATX* expression and overall *ATX* methylation level (β coefficient: -0.053, 95% CI: -0.072 to -0.035, *P*<0.0001) and also each of the four CpG sites, as follows: CpG 1 (β coefficient: -0.032, 95% CI: -0.044 to -0.020, *P*<0.0001), CpG 2 (β coefficient: -0.050, 95% CI: -0.067 to -0.033, *P*<0.0001), CpG 3 (β coefficient: -0.049, 95% CI: -0.069 to -0.028, *P*<0.0001), and CpG 4 (β coefficient: -0.043, 95% CI: -0.066 to -0.019, *P* = 0.001) (Table 3). Subsequent analysis revealed that serum ATX levels were correlated with biochemical parameters in BA patients (Table 5).

**Table 5:** Spearman's correlation and multivariate linear regression analysis of serum ATX level estimates.

Variables	Serum ATX levels (ng/ml)			
	Spearman's rho correlation		Linear regression^a^	
	Coefficient (*r*)	*P*-value	β coefficient (95% CI)	*P*-value
**Age (years)**	-0.04	0.77	-6.43 (-36.85 to 23.99)	0.67
**Liver stiffness (kPa)**	0.71	<0.0001*	15.77 (11.13 to 20.42)	<0.0001*
**TB (mg/dl)**	0.63	<0.0001*	83.18 (49.06 to 117.30)	<0.0001*
**AST (IU/l)**	0.77	<0.0001*	5.15 (3.90 to 6.39)	<0.0001*
**ALT (IU/l)**	0.53	<0.0001*	3.15 (1.54 to 4.75)	<0.0001*
**ALP (IU/l)**	0.68	<0.0001*	1.46 (1.00 to 1.93)	<0.0001*
**Albumin (g/dl)**	-0.68	<0.0001*	-285.32 (-470.00 to -99.93)	0.003*
***ATX* methylation levels (%)**				
** Overall**	-0.55	<0.0001*	-33.80 (-46.42 to -21.18)	<0.0001*
** CpG 1**	-0.61	<0.0001*	-21.11 (-29.17 to -12.95)	<0.0001*
** CpG 2**	-0.46	<0.0001*	-29.86 (-41.63 to -18.09)	<0.0001*
** CpG 3**	-0.34	0.006*	-28.66 (-43.14 to -14.18)	<0.0001*
** CpG 4**	-0.42	0.001*	-28.64 (-44.38 to -12.91)	0.0001*
**Relative mRNA expression (*ATX*/*GAPDH*)**	0.44	<0.0001*	288.60 (129.36 to 447.85)	0.001*

*Correlation is considered statistically significant at *P*-value less than 0.05 (two-tailed)

^a^The coefficient was adjusted for age and gender.

Abbreviations: TB = total bilirubin; AST = aspartate aminotransferase; ALT = alanine aminotransferase; ALP = Alkaline phosphatase

### *ATX* promoter hypomethylation increased its expression in liver tissue samples

**Fig 4:**
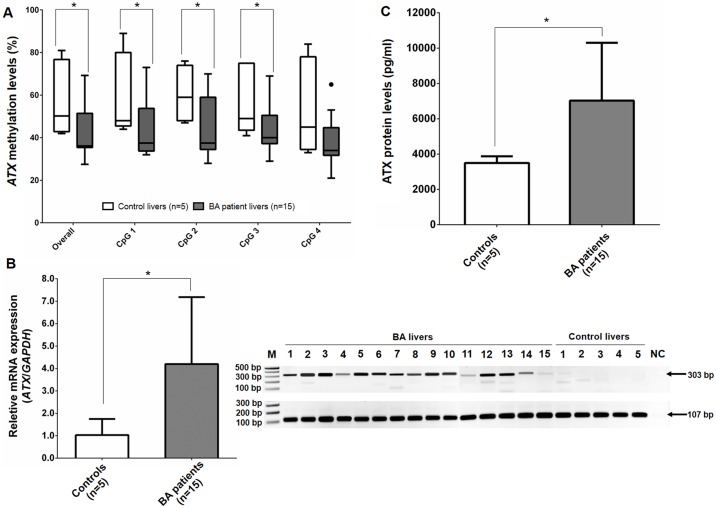
Distribution of the *ATX* promoter methylation, relative mRNA expression, and protein levels in liver tissue of BA patients and controls. (A) Decreased methylation levels of the *ATX* promoter in BA liver tissue samples. (B) Higher mRNA expression of *ATX* in BA cases and representative gel of *ATX* and *GAPDH* products from real-time PCR analysis. (C) Elevated ATX levels in liver tissue of BA patients. M, molecular weight marker, and NC, negative control. *P<0.05 vs control group.

To determine whether *ATX* promoter methylation in genomic DNA reflects epigenetic alterations in liver tissues, we examined methylation levels of the *ATX* promoter in the liver samples of 15 BA patients, compared with those of 5 non-BA controls. The total methylation status was significantly lower in BA livers than in control livers (*P* = 0.033), consistent with methylation levels in peripheral blood leukocytes. The three CpG sites at the *ATX* promoter showed less methylation in BA livers, when compared to control livers (CpG 1: *P* = 0.033, CpG 2: *P* = 0.045, and CpG 3: *P* = 0.047, respectively), but there were no significant differences in methylation levels of the *ATX* promoter at CpG 4 in either group (Fig 4A).

Further analysis showed that *ATX* mRNA expression levels in BA livers were markedly higher than those in non-BA control livers. In quantitative real-time PCR, we also observed a significantly higher *ATX* expression in the BA livers than in the control livers (*P*<0.05) (Fig 4B). Moreover, ATX protein levels were significantly higher in the BA livers, when compared to the non-BA control livers (*P*<0.05) (Fig 4C).

### Upregulated *DNMT1* expression in biliary atresia

**Fig 5:**
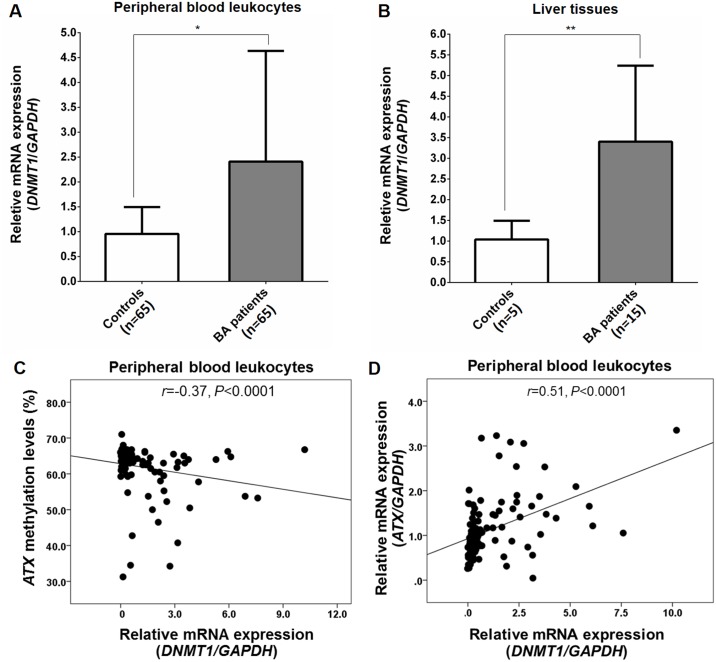
*DNMT1* mRNA expression in peripheral blood leukocytes and livers of BA patients and controls. (A) Relative *DNMT1* mRNA expression in peripheral blood leukocytes from BA patients and healthy controls. (B) Relative *DNMT1* mRNA expression in liver tissue samples from BA patients and healthy controls. (C) A negative correlation between relative *DNMT1* mRNA expression and *ATX* methylation in peripheral blood leukocytes from BA patients. (D) A positive correlation between relative *DNMT1* mRNA expression and relative *ATX* mRNA expression in peripheral blood leukocytes from BA patients. Data are expressed as mean and standard deviation. *P<0.05, **P<0.01 vs control group.

To determine *DNMT1* expression that may be responsible for *ATX* promoter methylation, we conducted quantitative real-time PCR for *DNMT1* in peripheral blood leukocytes and liver tissue samples. Quantitative real-time PCR showed that relative *DNMT1* mRNA expression in peripheral blood leukocytes was significantly higher in BA patients than healthy controls (*P*<0.05) (Fig 5A). There were no significant differences in relative *DNMT1* expression between early stage BA patients (non-jaundice, mild fibrosis, and low AST value) and advanced BA patients (jaundice, severe fibrosis, and high AST value) (*P*>0.05). Furthermore, relative *DNMT1* mRNA expression in BA livers was significantly increased when compared with that in control livers as shown in Fig 5B (*P*<0.01). No correlation between relative *DNMT1* expression and *ATX* methylation status was found in BA livers. Subsequent analysis illustrated that *DNMT1* mRNA expression was inversely correlated with *ATX* methylation (*r* = -0.37, *P*<0.0001) (Fig 5C) but *DNMT1* mRNA expression was positively correlated with *ATX* mRNA expression in peripheral blood leukocytes of BA patients (*r* = 0.51, *P*<0.0001) (Fig 5D).

### *ATX* promoter hypomethylation as a possible biomarker

**Fig 6:**
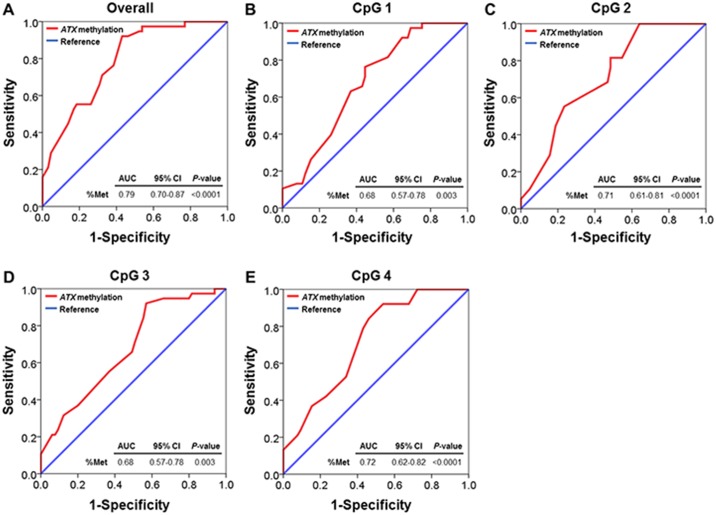
Receiver operating characteristic (ROC) curve representing the cutoff value of *ATX* promoter methylation in BA patients. (A) Overall *ATX* promoter methylation. (B) *ATX* promoter methylation at CpG 1 site. (C) *ATX* promoter methylation at CpG 2 site. (D) *ATX* promoter methylation at CpG 3 site. (E) *ATX* promoter methylation at CpG 4 site.

Additionally, we calculated the area under curve of the ROC curve, which was constructed using *ATX* methylation values. Based on the ROC curve, the optimal cutoff values of *ATX* methylation for overall, CpG 1, CpG 2, CpG 3, and CpG 4 as a possible biomarker for discriminating BA patients were projected to be 63.63, 63.50, 62.50, 63.50, and 63.50, respectively, which yielded the sensitivity of 81.60%, 63.20%, 81.60%, 65.80%, and 84.20% and the specificity of 60.00%, 63.10%, 51.60%, 50.80%, and 53.80%, respectively. The AUC of *ATX* methylation for overall, CpG 1, CpG 2, CpG 3, and CpG 4 were 0.79 (95% CI: 0.70 to 0.87, *P*<0.0001), 0.68 (95% CI: 0.57 to 0.78, *P* = 0.003), 0.71 (95% CI: 0.61 to 0.81, *P*<0.0001), 0.68 (95% CI: 0.57 to 0.78, *P* = 0.003), as well as 0.72 (95% CI: 0.62 to 0.82, *P*<0.0001), respectively (Fig 6A–6E).

## Discussion

The findings of this study add to an emerging body of literature that has investigated and reported on the molecular processes in peripheral blood leukocytes and liver tissues of individuals affected by biliary atresia. In this study, we demonstrated the presence of reduced DNA methylation at four CpGs within the promoter region of *ATX* in peripheral blood leukocytes and liver tissues of BA individuals. We also found that DNA hypomethylation of the *ATX* promoter might be responsible for elevated *ATX* mRNA expression. Notably, *ATX* expression was significantly more abundant in BA patients than in controls. In addition, upregulated *ATX* expression was observed in BA patients with fibrosis, as compared to BA patients without fibrosis. Data from our previous study provided evidence that increased serum ATX levels in BA patients were significantly correlated with the severity of BA [23]. We demonstrated the presence of higher *ATX* expression and protein levels in liver tissues of BA patients. Taken together, we found that higher *ATX* mRNA expression and protein levels were inversely correlated with hypomethylation of the gene promoter. The present study provides further evidence of the primary role that epigenetic modifications assume in influencing level of gene expression in biliary atresia.

To the best of our knowledge, this is the first study to report data regarding the potential epigenetic regulation of the *ATX* gene. We showed that specific CpGs within the *ATX* promoter were hypomethylated in BA patients, which was supported by significantly elevated *ATX* expression and a corresponding increase in ATX protein levels. To validate these findings, we also compared DNA methylation in BA livers with control livers and found CpGs within the *ATX* promoter to have lower methylated DNA in BA livers, which is consistent with the findings observed in peripheral blood leukocytes. The *ATX* promoter hypomethylation correlated negatively with the severity of clinical parameters and hepatic fibrosis (TB, AST, ALP, and liver stiffness) but positively with hepatic protein synthesis (albumin), suggesting that epigenetic mechanisms could play a possible role in the regulation of *ATX* expression regarding hepatic dysfunction and/or hepatic fibrosis. It seems plausible that the selected promoter regions of *ATX* comprise the coding sequence, thus affecting the transcription of the *ATX* gene. However, there was no relationship between *ATX* methylation at the CpG 4 residue and clinical outcome in BA. We speculate that the CpG 4 residue might not contain the coding sequence related to the transcription of the *ATX* gene, leading to no association of *ATX* promoter methylation at the CpG 4 site with outcome parameters in postoperative BA patients.

The current study also revealed that *DNMT1* mRNA expression in peripheral blood leukocytes and liver tissues was significantly elevated in BA patients compared with that in the controls. Further analysis showed that *DNMT1* mRNA expression was negatively associated with *ATX* methylation status but *DNMT1* mRNA expression was positively associated with *ATX* mRNA expression in peripheral blood leukocytes. The explanation for increased *DNMT1* mRNA expression in BA remains obscure. It might be related to the spectrum of the tested DNMTs and the detection method. The enzymatic activity of DNMT1 is controlled by both posttranscriptional and posttranslational mechanism [24]. In the present study, we investigate the association between *ATX* methylation status and *DNMT1* expression, but not with the enzymatic activities. Therefore, it is likely that the activity (and not the amount) of the enzyme is more important in BA. Additional research will be needed to determine the association of enzymatic activity of DNMT1 with the *ATX* methylation status.

Subsequent analysis demonstrated that BA patients had a significantly lower *ATX* methylation status and higher expression of *DNMT1* mRNA. The elevated *DNMT1* mRNA expression involved in *ATX* hypomethylation in BA patients is still unclear. DNA hypomethylation and higher expressions of DNMTs have been observed in chronic hepatitis, cirrhosis, and hepatocellular carcinoma [25]. This suggested a feedback mechanism of DNMTs on the methylation status. The increased *DNMT1* mRNA expression could be attributed to an indirect response to *ATX* hypomethylation in BA patients. It is postulated that the DNMT activity is itself regulated partially by DNA methylation status which represent a feedback mechanism. Accordingly, we suggest that *ATX* promoter methylation might depend upon several factors, including *DNMT* mRNA expression, DNMT activity, DNMT protein expression, and transcript levels of other enzymes involved in the DNA methylation. Furthermore, the promoter methylation and expression of autotaxin could depend on multiple pathways and molecules except the DNMT1 function only.

Our findings support prior evidence that DNA methylation is an important determinant of BA [15], as well as a stimulator of stellate cells and progressive hepatic fibrosis in animal models [26]. Nevertheless, no direct investigation of gene-specific methylation involving liver fibrosis in BA has been demonstrated. ATX has been shown to affect hepatic fibrogenesis, which has been implicated in the pathogenesis of liver fibrosis in BA, especially the stimulation of hepatic stellate cell proliferation via its enzymatic product, LPA [27]. Since serum levels of ATX and LPA have been correlated with the development of liver fibrosis [12], upregulated *ATX* expression might be associated with the severity of BA. In addition, our study showed significantly higher liver *ATX* mRNA expression in BA patients than in controls. This is consistent with previously reported observations demonstrating that ATX was more highly expressed in liver tissue of patients with HCC [28]. Wu *et al*. reported extensive evidence of *ATX* overexpression associated with progression of inflammation and liver cirrhosis in HCC patients [14], which supports our findings of upregulated *ATX* expression in BA patients with severe fibrosis. In recent years, elevated circulating ATX has been documented in patients with other liver diseases including chronic hepatitis C virus (HCV) infection, HCV-associated fibrosis, and cirrhosis [7, 11, 12]. Moreover, serum ATX was increased in hepatocellular carcinoma with liver fibrosis, nonalcoholic fatty liver disease, and cholestatic disorders [8, 29, 30]. ATX expression was also shown to be augmented in chronic cholestatic diseases such as primary biliary cholangitis and primary sclerosing cholangitis [31]. These findings lead us to hypothesize that ATX might serve as a possible parameter reflecting the severity of liver diseases including biliary atresia.

Currently, BA is accepted as a heterogeneous disease, with various forms of clinical presentation. The clinical manifestation of postoperative BA patients may reveal striking heterogeneity with a spectrum ranging from mild cases with early stages of liver fibrosis to severe cases with advanced stages of liver fibrosis. In the present study, promoter hypomethylation and overexpression of *ATX* varied between BA patients and, perhaps, between different stage of liver fibrosis. The possible explanation for this observation could be that *ATX* mRNA expression might be lower in the early stage liver fibrosis, and *ATX* expression may continuously increase during the disease progression. Additionally, the variation of hepatic *ATX* expression could be ascribed to the heterogeneity of liver fibrosis, with different stages being present in different areas of the BA livers. The etiology of BA remains elusive and theories of pathogenesis include viral infection, defects in bile duct development, genetics, and toxic factors [2–4]. It is also tempting to speculate that epigenetic factors may modulate the phenotypic manifestations of BA. Moreover, a number of growth factors and cytokines involved in the liver fibrosis remain largely unexplored. Autotaxin may act in concert with many other pro-inflammatory and profibrogenic cytokines and growth factors, which contribute to the liver fibrogenesis in BA.

Although the precise origin and fate of elevated circulating ATX levels remains unknown, both human and animal studies suggest that ATX is metabolized by the liver [10]. High serum ATX might result from decreased ATX clearance, increased expression, or a combination of both. A reduction in ATX clearance may result from diminished uptake by liver sinusoidal endothelial cells [32]. Yet ATX activity in liver disease is closely related with liver function, as ATX clearance is impaired when liver function failed in the case of BA, and other liver diseases [8, 10]. This mechanism may result in the high expression levels of ATX in BA. In the present study, we also found a positive association between mRNA expression and circulating protein levels of ATX in BA patients. Thus, it is speculated that a factor capable of increasing ATX expression (or reducing its clearance) in BA may result from phenotypic changes in liver sinusoidal endothelial cells during liver fibrosis.

This study suggests the possible involvement of ATX on liver fibrosis in BA. The aberrant production of ATX may lead to the altered activation of LPA signal transductions through G-protein coupled LPA receptors including, but not limited to, activation of Rho, Ras, phosphoinositide 3-kinase (PI3K) signaling pathways. LPA was shown to stimulate the proliferation and contraction of hepatic stellate cells and inhibit the apoptosis of these cells *in vitro* through Rho/Rho kinase activation, suggesting that LPA could be profibrogenic in liver [10]. LPA acts on its own G-protein coupled receptors and thereby elicit multiple cellular responses, such as Rho/Rac-regulated cell migration, Ras-mediated cell proliferation, and PI3K-mediated cell survival [33]. Accordingly, the aberrant expression of ATX along with the consequently abnormal production of LPA in the liver microenvironment may fuel the progression of liver fibrosis in biliary atresia.

It should be noted, however, that we are aware of some inherent limitations. First, the cross-sectional design prevents determination of causal relationships, and the potential for confounding variables cannot be dismissed. To further address this question of cause or consequence, prospective cohort or experimental studies will help to elucidate the effect of epigenetic changes on *ATX* expression and the risk of liver fibrosis in BA. Secondly, the number of cases and controls in our study was relatively small, especially regarding the effect of epigenetic changes in ATX produced in liver tissues. This factor diminishes the statistical power and generalizability of our results. It is recommended that future research should be conducted on a large scale using multicenter studies to further address how increased *ATX* expression may influence the pathogenesis in BA patients. Lastly, incomplete assessment of possible confounding variables including medical comorbidities needs to be taken into consideration.

## Conclusion

This study revealed that hypomethylation of CpG islands within the promoter region of the *ATX* gene was associated with overexpression of *ATX* mRNA and protein levels, which might play a plausible role in the pathogenesis of liver fibrosis in BA. Our results demonstrate a possible correlation between *ATX* methylation and levels of *ATX* transcription and translation. These findings imply that the epigenetic aberrance of *ATX* promoter hypomethylation status might contribute to liver fibrosis and has been hypothesized to be a potential biomarker for monitoring the progression of liver fibrosis in postoperative biliary atresia. Further validation with prospective studies is necessary to determine the utility of ATX as a biomarker for the risk of liver fibrosis in BA. Given the established role of ATX in promoting liver fibrosis, additional studies of potential underlying mechanisms related to the effect of ATX on the pathogenesis of liver fibrosis in BA are warranted.

## Supporting Information

S1 Table *ATX* promoter methylation distribution in the study participants. (DOC)

## Abbreviations

ALP: alkaline phosphatase
ALT: alanine aminotransferase
AST: aspartate aminotransferase
ATX: autotaxin
AUC: area under the ROC curve
BA: biliary atresia
CI: confidence interval
CpG: cytosine–guanine dinucleotide
DNMTs: DNA methyltransferases
ECM: extracellular matrix
ELISA: enzyme-linked immunosorbent assay
ENPP: ectonucleotide pyrophosphatase/ phosphodiesterase
GAPDH: glyceraldehyde 3-phosphate dehydrogenase
HCC: hepatocellular carcinoma
HCV: hepatitis C virus
HSC: hepatic stellate cell
IFN-γ: interferon gamma
kPa: kilopascals
LPA: lysophosphatidic acid
LPC: lysophosphatidylcholine
LPD: lysophospholipase D
OR: odds ratio
PCR: polymerase chain reaction
QPCR: quantitative real-time polymerase chain reaction
ROC: Receiver operating characteristic
TB: total bilirubin
