# Supplementary material for: Association between Promoter Hypomethylation and Overexpression of Autotaxin with Outcome Parameters in Biliary Atresia
Source: PLoS One. 2017 Jan 4;12(1):e0169306. doi: 10.1371/journal.pone.0169306 (PMC5214988; doi:10.1371/journal.pone.0169306)
Supplement: S1 Table — (DOC) [file pone.0169306.s001.doc]

**Supporting information**

**S1 Table.** *ATX* promoter methylation distribution in the study participants

| **No.** | **CpG 1** | **CpG 2** | **CpG 3** | **CpG 4** | **Overall** |
| --- | --- | --- | --- | --- | --- |
| **Control ( C ) leukocytes** | | | |  |  |
| C1 | 65 | 68 | 62 | 60 | 63.75 |
| C2 | 55 | 66 | 67 | 68 | 64 |
| C3 | 59 | 60 | 70 | 65 | 63.5 |
| C4 | 64 | 64 | 61 | 65 | 63.5 |
| C5 | 61 | 63 | 65 | 63 | 63 |
| C6 | 65 | 68 | 62 | 63 | 64.5 |
| C7 | 59 | 60 | 63 | 65 | 61.75 |
| C8 | 66 | 75 | 60 | 66 | 66.75 |
| C9 | 62 | 65 | 70 | 64 | 65.25 |
| C10 | 64 | 67 | 64 | 68 | 65.75 |
| C11 | 64 | 67 | 63 | 68 | 65.5 |
| C12 | 47 | 60 | 46 | 66 | 54.75 |
| C13 | 63 | 65 | 66 | 64 | 64.5 |
| C14 | 64 | 60 | 64 | 66 | 63.5 |
| C15 | 64 | 68 | 65 | 69 | 66.5 |
| C16 | 60 | 65 | 70 | 60 | 63.75 |
| C17 | 60 | 64 | 55 | 65 | 61 |
| C18 | 70 | 60 | 70 | 65 | 66.25 |
| C19 | 70 | 67 | 62 | 65 | 66 |
| C20 | 62 | 65 | 64 | 67 | 64.5 |
| C21 | 65 | 68 | 62 | 66 | 65.25 |
| C22 | 66 | 67 | 66 | 67 | 66.5 |
| C23 | 70 | 60 | 58 | 65 | 63.25 |
| C24 | 59 | 60 | 60 | 60 | 59.75 |
| C25 | 66 | 67 | 64 | 65 | 65.5 |
| C26 | 64 | 66 | 65 | 60 | 63.75 |
| C27 | 61 | 68 | 61 | 64 | 63.5 |
| C28 | 57 | 65 | 67 | 65 | 63.5 |
| C29 | 70 | 67 | 55 | 68 | 65 |
| C30 | 64 | 62 | 65 | 69 | 65 |
| C31 | 64 | 60 | 63 | 66 | 63.25 |
| C32 | 67 | 66 | 65 | 68 | 66.5 |
| C33 | 54 | 70 | 70 | 56 | 62.5 |
| C34 | 59 | 71 | 68 | 63 | 65.25 |
| C35 | 63 | 72 | 70 | 62 | 66.75 |
| C36 | 70 | 72 | 71 | 71 | 71 |
| C37 | 50 | 65 | 67 | 57 | 59.75 |
| C38 | 57 | 70 | 70 | 63 | 65 |
| C39 | 56 | 70 | 71 | 60 | 64.25 |
| C40 | 57 | 73 | 68 | 61 | 64.75 |
| C41 | 45 | 70 | 70 | 69 | 63.5 |
| C42 | 70 | 69 | 70 | 55 | 66 |
| C43 | 63 | 70 | 63 | 62 | 64.5 |
| C44 | 70 | 61 | 70 | 63 | 66 |
| C45 | 63 | 70 | 71 | 60 | 66 |
| C46 | 63 | 68 | 69 | 61 | 65.25 |
| C47 | 61 | 70 | 70 | 53 | 63.5 |
| C48 | 66 | 67 | 67 | 61 | 65.25 |
| C49 | 49 | 67 | 65 | 66 | 61.75 |
| C50 | 61 | 62 | 70 | 70 | 65.75 |
| C51 | 64 | 69 | 70 | 69 | 68 |
| C52 | 70 | 70 | 61 | 61 | 65.5 |
| C53 | 64 | 61 | 60 | 59 | 61 |
| C54 | 62 | 70 | 70 | 60 | 65.5 |
| C55 | 56 | 72 | 67 | 65 | 65 |
| C56 | 64 | 67 | 67 | 55 | 63.25 |
| C57 | 62 | 70 | 70 | 64 | 66.5 |
| C58 | 64 | 66 | 73 | 65 | 67 |
| C59 | 63 | 70 | 70 | 62 | 66.25 |
| C60 | 52 | 67 | 65 | 67 | 62.75 |
| C61 | 64 | 67 | 67 | 65 | 65.75 |
| C62 | 47 | 70 | 70 | 50 | 59.25 |
| C63 | 52 | 65 | 67 | 62 | 61.5 |
| C64 | 65 | 70 | 66 | 66 | 66.75 |
| C65 | 63 | 67 | 67 | 62 | 64.75 |
| **Biliary atresia (BA) leukocytes** | | | |  |  |
| BA1 | 40 | 55 | 58 | 49 | 50.5 |
| BA2 | 50 | 60 | 65 | 67 | 60.5 |
| BA3 | 65 | 65 | 64 | 68 | 65.5 |
| BA4 | 65 | 65 | 60 | 62 | 63 |
| BA5 | 64 | 62 | 65 | 69 | 65 |
| BA6 | 59 | 59 | 60 | 59 | 59.25 |
| BA7 | 49 | 57 | 60 | 65 | 57.75 |
| BA8 | 60 | 68 | 65 | 60 | 63.25 |
| BA9 | 33 | 58 | 58 | 66 | 53.75 |
| BA10 | 43 | 57 | 60 | 53 | 53.25 |
| BA11 | 65 | 59 | 64 | 68 | 64 |
| BA12 | 65 | 66 | 67 | 62 | 65 |
| BA13 | 68 | 65 | 64 | 62 | 64.75 |
| BA14 | 64 | 60 | 64 | 68 | 64 |
| BA15 | 63 | 59 | 66 | 64 | 63 |
| BA16 | 58 | 57 | 60 | 63 | 59.5 |
| BA17 | 35 | 40 | 55 | 56 | 46.5 |
| BA18 | 46 | 56 | 50 | 63 | 53.75 |
| BA19 | 60 | 62 | 66 | 62 | 62.5 |
| BA20 | 24 | 35 | 36 | 42 | 34.25 |
| BA21 | 40 | 49 | 54 | 57 | 50 |
| BA22 | 62 | 65 | 65 | 66 | 64.5 |
| BA23 | 59 | 62 | 60 | 61 | 60.5 |
| BA24 | 25 | 37 | 45 | 56 | 40.75 |
| BA25 | 64 | 68 | 66 | 67 | 66.25 |
| BA26 | 66 | 65 | 64 | 63 | 64.5 |
| BA27 | 33 | 40 | 43 | 55 | 42.75 |
| BA28 | 68 | 65 | 69 | 62 | 66 |
| BA29 | 64 | 60 | 58 | 66 | 62 |
| BA30 | 60 | 65 | 70 | 49 | 61 |
| BA31 | 39 | 68 | 70 | 70 | 61.75 |
| BA32 | 66 | 67 | 65 | 67 | 66.25 |
| BA33 | 64 | 50 | 62 | 70 | 61.5 |
| BA34 | 65 | 70 | 65 | 65 | 66.25 |
| BA35 | 23 | 38 | 35 | 42 | 34.5 |
| BA36 | 70 | 70 | 57 | 70 | 66.75 |
| BA37 | 67 | 68 | 70 | 62 | 66.75 |
| BA38 | 49 | 59 | 54 | 59 | 55.25 |
| BA39 | 64 | 65 | 63 | 64 | 64 |
| BA40 | 17 | 37 | 48 | 23 | 31.25 |
| BA41 | 64 | 66 | 65 | 68 | 65.75 |
| BA42 | 45 | 57 | 59 | 48 | 52.25 |
| BA43 | 55 | 60 | 62 | 55 | 58 |
| BA44 | 63 | 70 | 64 | 65 | 65.5 |
| BA45 | 70 | 62 | 65 | 67 | 66 |
| BA46 | 59 | 65 | 58 | 70 | 63 |
| BA47 | 36 | 40 | 47 | 38 | 40.25 |
| BA48 | 70 | 60 | 61 | 63 | 63.5 |
| BA49 | 63 | 66 | 64 | 66 | 64.75 |
| BA50 | 60 | 65 | 64 | 66 | 63.75 |
| BA51 | 65 | 68 | 62 | 66 | 65.25 |
| BA52 | 70 | 65 | 68 | 63 | 66.5 |
| BA53 | 56 | 50 | 56 | 60 | 55.5 |
| BA54 | 63 | 65 | 59 | 66 | 63.25 |
| BA55 | 34 | 48 | 53 | 54 | 47.25 |
| BA56 | 70 | 64 | 49 | 60 | 60.75 |
| BA57 | 50 | 65 | 67 | 65 | 61.75 |
| BA58 | 60 | 60 | 65 | 55 | 60 |
| BA59 | 60 | 68 | 70 | 62 | 65 |
| BA60 | 65 | 60 | 66 | 67 | 64.5 |
| BA61 | 60 | 65 | 66 | 65 | 64 |
| BA62 | 59 | 68 | 65 | 62 | 63.5 |
| BA63 | 60 | 67 | 65 | 65 | 64.25 |
| BA64 | 60 | 65 | 64 | 66 | 63.75 |
| BA65 | 64 | 66 | 65 | 68 | 65.75 |
| **Control ( C ) livers** | | |  |  |  |
| C1 | 48 | 59 | 49 | 45 | 50.25 |
| C2 | 44 | 47 | 41 | 36 | 42 |
| C3 | 47 | 49 | 46 | 33 | 43.75 |
| C4 | 62 | 68 | 52 | 53 | 58.75 |
| C5 | 40 | 56 | 40 | 38 | 43.5 |
| **Biliary atresia (BA) livers** | | | |  |  |
| BA1 | 39 | 33 | 35 | 31 | 34.5 |
| BA2 | 33 | 37 | 40 | 35 | 36.25 |
| BA3 | 71 | 72 | 75 | 72 | 72.5 |
| BA4 | 34 | 35 | 42 | 33 | 36 |
| BA5 | 100 | 76 | 75 | 84 | 83.75 |
| BA6 | 51 | 53 | 50 | 42 | 49 |
| BA7 | 73 | 70 | 69 | 65 | 69.25 |
| BA8 | 32 | 28 | 29 | 21 | 27.5 |
| BA9 | 36 | 37 | 38 | 32 | 35.75 |
| BA10 | 40 | 56 | 40 | 38 | 43.5 |
| BA11 | 62 | 68 | 52 | 53 | 58.75 |
| BA12 | 34 | 38 | 38 | 33 | 35.75 |
| BA13 | 29 | 25 | 27 | 21 | 24.5 |
| BA14 | 34 | 28 | 30 | 31 | 30.75 |
| BA15 | 39 | 55 | 39 | 37 | 42.5 |
